# Transposon Excision from an Atypical Site: A Mechanism of Evolution of Novel Transposable Elements

**DOI:** 10.1371/journal.pone.0000965

**Published:** 2007-10-03

**Authors:** Marybeth Langer, Lynn F. Sniderhan, Ueli Grossniklaus, Animesh Ray

**Affiliations:** 1 Department of Biology, University of Rochester, Rochester, New York, United States of America; 2 Cold Spring Harbor Laboratory, Cold Spring Harbor, New York, United States of America; 3 Keck Graduate Institute, Claremont, California, United States of America; University of California at Davis, United States of America

## Abstract

The role of transposable elements in sculpting the genome is well appreciated but remains poorly understood. Some organisms, such as humans, do not have active transposons; however, transposable elements were presumably active in their ancestral genomes. Of specific interest is whether the DNA surrounding the sites of transposon excision become recombinogenic, thus bringing about homologous recombination. Previous studies in maize and Drosophila have provided conflicting evidence on whether transposon excision is correlated with homologous recombination. Here we take advantage of an atypical *Dissociation* (*Ds*) element, a maize transposon that can be mobilized by the *Ac* transposase gene in *Arabidopsis thaliana*, to address questions on the mechanism of *Ds* excision. This atypical *Ds* element contains an adjacent 598 base pairs (bp) inverted repeat; the element was allowed to excise by the introduction of an unlinked *Ac* transposase source through mating. Footprints at the excision site suggest a micro-homology mediated non-homologous end joining reminiscent of V(D)J recombination involving the formation of intra-helix 3′ to 5′ trans-esterification as an intermediate, a mechanism consistent with previous observations in maize, Antirrhinum and in certain insects. The proposed mechanism suggests that the broken chromosome at the excision site should not allow recombinational interaction with the homologous chromosome, and that the linked inverted repeat should also be mobilizable. To test the first prediction, we measured recombination of flanking chromosomal arms selected for the excision of *Ds*. In congruence with the model, *Ds* excision did not influence crossover recombination. Furthermore, evidence for correlated movement of the adjacent inverted repeat sequence is presented; its origin and movement suggest a novel mechanism for the evolution of repeated elements. Taken together these results suggest that the movement of transposable elements themselves may not directly influence linkage. Possibility remains, however, for novel repeated DNA sequences produced as a consequence of transposon movement to influence crossover in subsequent generations.

## Introduction

Mobile genetic elements are important forces of evolution [1]. *Ac/Ds* elements are DNA transposons that use a conservative “cut-and-paste” mechanism involving double-stranded DNA breaks to move within the genome. Repair of broken chromosome ends at the donor (excision) site contributes to genetic alterations in adjoining sequences [2]–[4]. Footprints or DNA sequence rearrangements left at the donor site following transposition provide clues to the repair mechanisms [3], [5], [6].

The exact mechanism of donor site repair remains unresolved; indeed, it appears likely that this phenomenon may be affected by host proteins and hence might vary among species [3]. In particular the effect of *Ac/Ds* transposition on chromosome crossover, a major mechanism behind genetic diversity [7], is an unanswered question. Linkage disequilibrium, the historical evidence of rare crossover exchanges between close markers, may potentially have its origin, among other factors, in rare crossovers stimulated by the activity of mobile elements. Early studies in maize of *Activator/Dissociation* (*Ac/Ds*) elements had failed to find a clear association between crossing over and transposition [8], [9]. Although transposons are frequently involved in gene conversion [10] and stimulate crossover within repeated DNA [11], recent genome level analysis shows either no correlation [12] or negative correlation [13], [14] between recombination rate and transposable element density along the chromosome. One may speculate that this may be the consequence of evolutionary selection to prevent crossover exchanges where transposon density is high. The question remains whether a pioneering transposable element in a chromosome free of prior copies of that transposon allows high frequency crossover during its movement.

To address these questions, here we examined transposition of an active *Ds* element artificially introduced into *Arabidopsis thaliana.* The *Ds* element has an atypical insertion site, in which there is a long unrelated inverted repeat DNA sequence located immediately adjacent to, but outside of, one of the terminal inverted repeats, which is bordered on the outside by one of the duplicated target sequences. As in previous reports [3], [15], [16] we show that the excision repair footprints support a mechanism resembling immunoglobulin V(D)J class switching in vertebrates [17], [18]. In accordance with the proposed model of *Ds* transposition, we show that transposition does not influence crossover recombination between homologous chromosomes. This result suggests that the movement of transposable elements may not be an important factor in genome rearrangement by recombination. Furthermore, as predicted by the model, we detected movement of the unrelated DNA inverted repeat from its initial position to secondary locations. These observations, along with an analysis of the footprints of transposition, provoke a new model of DNA repair at the excision site that allows the formation of new inverted repeat sequences adjacent to the transposable element. We speculate that such inverted repeats may subsequently separate from the original transposon and form novel mobile sequences.

## Results

### Origin of a DNA inverted repeat sequence associated with *Ds*

**Figure 1:**
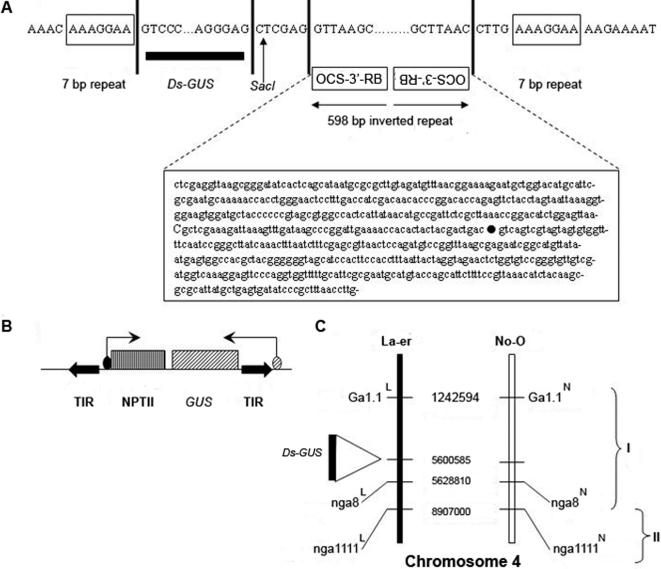
Structure and position of the *Ds* element in strain ET500. (A) Sequence of the *Ds* element and adjacent sequences showing the important elements. The insert is flanked by 7 bp target site duplication (boxed). DNA outside the duplicated target sequences are identical to the surrounding plant DNA sequence. Sequence of the 598 bp inverted repeat is identical to a fragment of the T-DNA containing 3′-octopine synthase promoter (3′OCS) and the right border (RB) element. Black circle within the boxed sequence shows the site of sequence inversion. (B) Structure of the *Ds* element. The *Ds* element contains a promoter-less bacterial *GUS* gene just inside the leftward TIR, and a bacterial *NPTII* gene with its own promoter. A plant-specific promoter-enhancer element (stippled oval) 5′ to the *Ds* insertion site confers constitutive *GUS* expression. (C) Map of the chromosomal region containing *Ds*. Numbers denote the first base on chromosome 4 (counting from the top) for the respective site. Roman numerals denote the two genetic intervals assayed for crossover. The transposase source, *Ac*, was unlinked to chromosome 4, and was present only in the experimental cross. Superscripts identify La-er (L) and No-0 (N) alleles.

The *Ds* site from which excision was allowed has an atypical configuration at the 3′ end: the 3′ terminal inverted repeat (3′ TIR) of the *Ds* end is situated adjacent to another inverted repeat of 598 bp (Figure 1A). The sequence of this repeat was derived from the parental strain [19] in which the maize *Ds* element, introduced into Arabidopsis on a T-DNA vector, had previously transposed into the current donor site. The presence of the 598 bp repeat suggests that this duplicated sequence transposed into this site from its original location on a T-DNA stably incorporated into the chromosome in its parent. (Figure 1B). Although the 5′ end of the *Ds* insertion has the typical half-site of a target duplication, the other target half-site is separated from the *Ds* TIR by the 598 bp inverted repeat sequence derived from the T-DNA vector. To test whether this modified *Ds* element can transpose, we mobilized the element by introducing an active *Ac* transposase gene as described below.

### Mobilization of the abnormal *Ds* element

**Figure 2:**
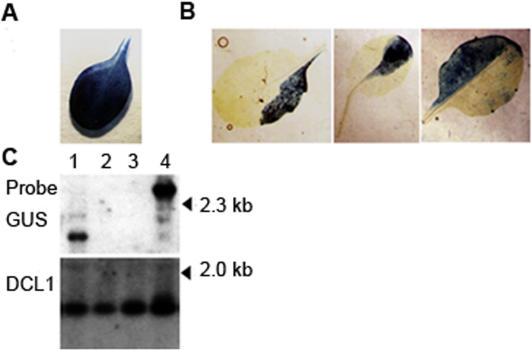
Excision of *Ds*. (A) A leaf of a parental *Ds* strain homozygous for *Ds* (ET500), showing uniform GUS^+^ staining. (B) GUS^−^ somatic sectors due to *Ds* excision in F1 of ET500×Nae*Ac*. (C) Southern blot analysis showing the excision of *Ds*. Upper panel was probed with GUS; lower panel represents the same blot cut off between 2kb and 3kb markers, and probed with DCL1 cDNA sequences as a loading control [19]. Lane 1, ET500 parental (before *Ds* excision). Lanes 2-3, DNA from two independent F2 derivatives of ET500×Nae*Ac* showing *Ds* excision, and lane 4, DNA from an F2 plant showing excision and reinsertion of *Ds* to another location. (D) Amplification of molecular markers: Landsberg *erecta* (L); Nossen (N); heterozygous (L/N) Left hand panel, nga8; middle panel, Ga1.1/BsaB1; right hand panel, nga1111.

We crossed two strains of *Arabidopsis thaliana*, Landsberg *erecta* (La-er) and Nossen (No-0) such that the F1 provided heterozygosities at molecular markers for scoring recombinants (Figure 1C). The experimental cross brought an enhancer detector *Ds* element from one parent together with an unlinked and immobile *Ac* transposase gene from the other. The *Ds* contains a beta-glucuronidase (*GUS*) reporter gene and a neomycin phosphotransferase (*NPTII*) marker located within bounds of the two TIRs (Figure 1B), while *Ac* is adjacent to the negatively-selectable *TMS2* gene encoding an amide hydrolase that imparts sensitivity to naphthalene acetamide [19]. The F1 of the corresponding control cross in the same genetic background carried the same *Ds* but not the *Ac*. The GUS reporter gene was under the control of an endogenous enhancer leading to constitutive expression such that production of GUS^−^ somatic sectors could serve as the hallmark of *Ds* excision. The control cross did not show any evidence of somatic *Ds* excision (32 F1 plants tested) as indicated by a lack of GUS^−^ leaf sectors (Figure 2A), nor of germinal excision as evidenced by transmission to the F2. By contrast, high frequency *Ds* excision occurred in 47 of 53 F1 plants of the experimental cross, as evidenced by GUS^−^ sectors on F1 leaves (Figure 2B) and confirmed by polymerase chain reaction (PCR) and Southern analysis following transmission into the F2 progeny (Figure 2C). To prevent continued transposition during analysis, F2 plants were selected on naphthaline acetamide (NAM) to eliminate descendents inheriting *Ac*.

Depending on the timing and relative rates of *Ds* excision in the F1, meristem cells contributing to the germ line may contain numerous independent transposition events or one early event with numerous clonal descendents. Thus, a single F2 family from one flower could represent multiple independent excision events or one clonal event. A sampling of independent events could not be guaranteed by analyzing a number of F2's derived from a single F1 plant. To overcome this difficulty, from each F1 parent only one F2 progeny plant was scored for recombination. Each F2 plant was selected for analysis at random from among a pool of F2 plants that was proven to have an excision event by PCR analysis of the donor site and confirmed by Southern blots. Each separate pool was derived from a single F1 parent. This guaranteed that each F2 plant examined represented an independent *Ds* excision. Although it was not possible to formally distinguish between premeiotic, meiotic or postmeiotic transposition, most transposition probably occurred premeiotically because of the observed high rate of excision plants per F1 line. There was no attempt to determine whether crossover events were somatic or meiotic.

### Excision footprints suggest V(D)J end joining type of repair

**Figure 3:**
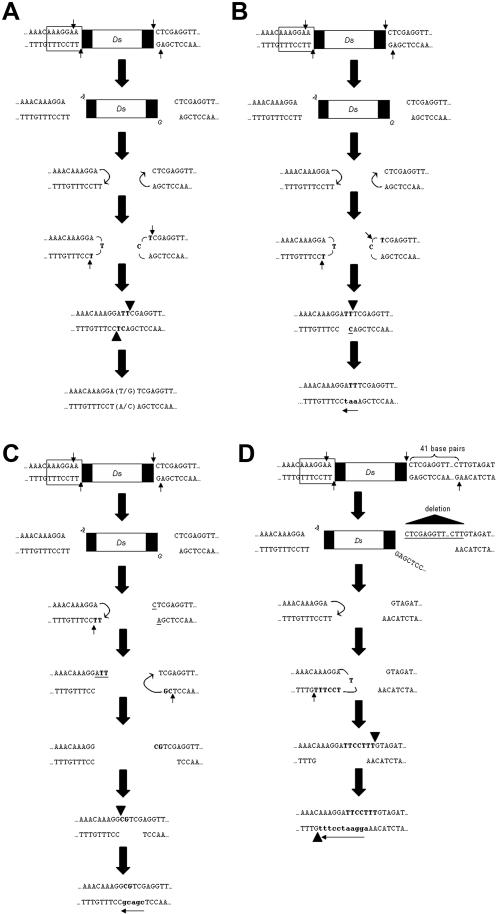
Models explaining *Ds* excision footprints. (A–C) A model explaining the formation of the most common footprints. Boxed bases are the 7bp duplicated target sequence. Thin straight arrows show putative transposase nick sites, curved arrows depict *trans*-esterification reaction between the 3′ OH group of one strand and the 5′ phosphate of the other, and arrowheads show the points of ligation. Gap repair synthesis bases are in bold and direction of synthesis is shown by an arrow; deleted bases are underlined. Note the absence of symmetrical targets flanking the excision site. (D) Model explaining the formation of a less common footprint.

**Figure 4:**
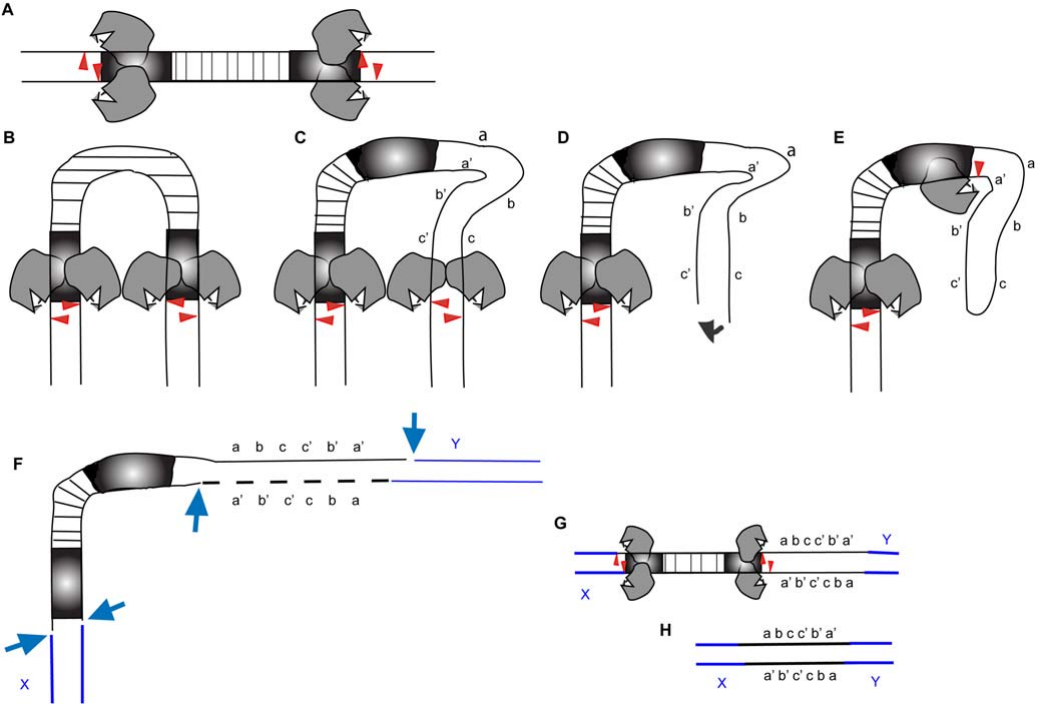
A model proposing the formation and movement of novel inverted repeat elements. (A) TIRs of a transposable element are shown in filled boxes. The boxed sequences are the 7bp duplicated half-sites. Transposase molecules bind at the TIRs, and (B) make pairs of staggered nicks on each flank (triangles). Occasionally, the DNA may slip (C) or the transposase may bind to cryptic sites on adjacent host DNA, and make a paired nick, which leads to excision (D). (E) Short repair synthesis followed by trans-esterification leads to a hairpin at the host segment attached to the TIR, which is nicked again near the TIR. Helix unwinding, followed by microhomology-mediated ligation to a new host site inserts the transposon, and single strand gap filling by repair synthesis forms a novel inverted repeat derived from host sequences adjacent to the original location (F). The transposon may repeat the cycle of precise (B) or imprecise (C) excision, and may be dissociated from novel host-derived inverted repeat (G). If the host-derived sequence had a cryptic transposase-binding site, the site is now duplicated on the novel inverted repeat. This may lead to independent movement of the inverted repeat sequence as a novel mobile element so long as a transposase is available.

DNA sequences at 11 of 17 independently derived empty *Ds* donor sites showed the same footprint and at three others a single base difference from the commonest footprint (Table 1). Sequences remaining at the excision sites were most consistent with the idea that excision frequently involved only one of the duplicated target sequences of the original *Ds* integration site. The footprints suggest a micro-homology mediated non-homologous end joining mechanism reminiscent of V(D)J recombination as suggested for Tam3 excision in *Antirrhinum* [15], *Ds* excision in maize [3], [16] and *Arabidopsis* [3], and shown for *in vitro* transposition of the insect *hAT* element *Hermes* [20] and *in vivo* excision of *Ds* in yeast [4], [21], involving pairs of staggered nicks, followed by DNA synthesis, hairpin formation by *trans*-esterification, hairpin nicking, imperfect base pairing, ligation, and mismatch correction (Figure 3A). Essentially similar models can explain two additional footprints (Figure 3B, C), and the remaining one involved a 41 base pairs deletion (Figure 3D). The exact site where the nick distal to the *Ds* element occurred cannot be ascertained from the footprint result; however, previous studies in maize, Arabidopsis and tobacco [3], [4], [15], [16], [22], [23] have determined that the footprints are most consistent with the transposase-mediated cleavage occurring at the 5′ nucleotide flanking the insertion. Our results are consistent with the previous models for 16 of 17 footprints (Figure 3). For the remaining footprint, involving a 41 bp deletion (Fig 4D), a mechanism involving an exonuclease-mediated degradation starting at a position 5′ of the Ds element is plausible, consistent with studies that show exonucleolytic deletion of sequences flanking the end of an element [24], [25]. However, the model we have proposed here (Fig. 3) explains all footprints observed here, including footprints reminiscent of V(D)J recombination, a footprint involving the 41 bp deletion and the formation of a 598 bp inverted repeat adjacent to the 3′ TIR of the starting *Ds* used in this study.

**Table 1:** Repair footprints following *Ds* excision

DNA sequence	Number of independent occurrences
…AAACAAAGGAA—*Ds*—CTCGAGGTT...	(Parental sequence before excision)
…AAACAAAGGATTCGAGGTT…	11
…AAACAAAGGAGTCGAGGTT…	3
…AAACAAAGGATTTCGAGGTT…	1
…AAACAAAGGCGTCGAGGTT…	1
…AAACAAAGGATTCCTTT…(41nt)…GTA…	1

The model suggests two independent predictions. First, the proposed mechanism of restoration of the broken chromosome does not involve any opportunity for homologous recombination initiated by the DNA double strand breaks because the broken ends are trans-esterified into hairpins, therefore, cannot initiate double strand break repair [26] without further processing. The second more subtle prediction concerns the observed 41 bp ‘sliding’ of the transposase to produce the second nick (Figure 3D). It is conceivable that a slide by the transposase could make a double chain break at a site distant from the 3′TIR, upon which the new end could undergo hairpin formation, followed by a second nick adjacent to the 3′TIR (Figure 4). As a result, a new inverted repeat sequence could form, as explained in Fig. 4. While this prediction is actually prompted by the observation of the 3′ inverted repeat at the original *Ds* element site (Figure 1A), it also suggests that a reversal of the process may occasionally co-mobilize the inverted repeat sequence along with the *Ds* element. Tests of these predictions are as follows.

### Flanking marker recombination with or without *Ds* excision

Among F2 seedlings from the experimental cross *Ac* (No-0)×*Ds* (La-er), we selected progeny that had experienced a *Ds* excision in the following way. We required that each F2 plant lacked *Ac*, was uniformly GUS^−^, and had at least one La-er allele of nga8 (nga8^L^) tightly linked to *Ds* (Figure 1C). These plants contained at least one chromosome from which *Ds* had excised from the donor site. Single plants representing independent events from each of 47 F1 families from the experimental cross were identified, and analysed by PCR for crossover within Ga1.1—nga8—nga1111 intervals (Figure 1C). Of 47 independent excision lines, 8 had crossover recombination between nga1111 and Ga1.1 (Table 2). This recombination rate (8.5% per chromosome) is not significantly different from 10% recombination per chromosome in the control cross where no transposition was possible (29 crossovers among 144 F2 plants tested). Given 10% spontaneous crossover rate, 17 or more crossovers per 47 *Ds* excision events would have been scored as significantly correlated (χ^2^ = 4.98; *P* ≤ 0.05, for 1 degree of freedom). Thus, the ability to detect an increase in crossover rate to 18% or higher argues that we would have detected a 50% crossover rate predicted by the double strand break-repair mechanism [26] had the repair following *Ds* excision occurred by the strict version of this mechanism. Crossover frequency below 18% is presumably due to events unrelated to *Ds* excision.

**Table 2:** *Ds* excision does not influence crossover recombination

Cross	Number of F1 families analyzed	Interval I (Ga1.1—nga8)		Interval II (nga8—nga1111)	
		Number of recombinants	Recombination frequency per chromosome	Number of recombinants	Recombination frequency per chromosome
*Ds*×*No-0*	144	23	7.9%	6	2.1%
*Ds*×*Ac*	47	6	6.4%	2	2.1%

A critical condition of the above test was that selection on NAM did not influence segregation of the three markers flanking *Ds*, namely, nga8, Ga1.1 and nga1111. We tested this by crossing the *Ac* (No-0) parent to wild type La-er, and analyzing marker segregation in F2 lines that were either selected or not selected on NAM. For 26 F2 plants not selected on NAM, the number of plants segregating homozygous Nossen alleles (*NN*): heterozygous (*NL*): homozygous Landsberg alleles (*LL*) were 9∶12∶5 for nga8, 6∶15∶5 for Ga1.1, and 8∶13∶5 for nga1111, respectively (see Figure 2D for a typical result). For those selected on NAM, the corresponding numbers were 9∶17∶1 for nga8, 11∶13∶3 for Ga1.1 and 8∶17∶2 for nga1111. The pairs of segregation ratios of the selected versus unselected plants were indistinguishable from each other by the chi-squared test (*P*≤0.2 for nga8; *P*≤1.0 for both Ga1.1 and nga111, respectively). In these crosses, crossover frequencies in Ga1.1—nga8 and nga8—nga1111 intervals were 3/52 chromosomes and 1/52 chromosomes, respectively, in the presence of NAM selection, and 4/54 chromosomes and 2/54 chromosomes, respectively, in the absence of NAM selection. These pairs of frequencies in selected versus unselected lines are statistically indistinguishable. Moreover, for 231 F2 progeny of 53 families screened for Ds excision (selection on NAM for *Ac* ^−^, GUS^−^ phenotype) the ratio of *LL*:*NL*:*NN* was 24%∶45%∶31%, showing no significant deficiency of any class with selection on NAM. The *TMS2* locus is unlinked to nga8, satisfying another requirement of the experiment. Linkage of TMS2 (*N*) to nga8*^N^* would have resulted in a deficiency of *NN* at nga8 with negative selection on NAM. PCR confirmed that NAM^S ^plants were also *Ac* ^−^.

### Correlated movement of unrelated inverted repeat

**Figure 5:**
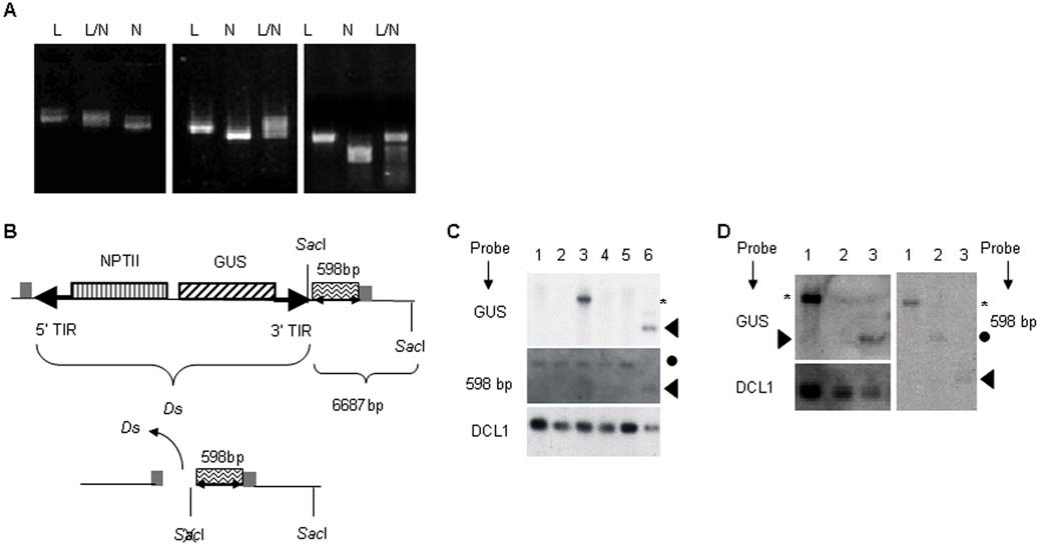
Excision of *Ds* with atypical flanking DNA. (A) Configuration of *Ds* in ET500 parental *Ds* element with *NPTII*, *GUS*, 5′ and 3′ TIRs. The 598bp inverted repeat is immediately 3′ of the *Ds* 3′ TIR. The duplicated target half sites (gray boxes) flank the 5′ TIR (typical) and the 598bp inverted repeat (atypical). (B) Excision of *Ds* yields broken ends that are adjacent to the leftward duplicated target at the 5′ end of *Ds* and to the 598bp inverted repeat at the 3′ end of *Ds*, and is predicted to destroy a *Sac*I site. (C) Southern blot analysis of plant DNA digested with *Sac*I, confirming *Ds* excision. Upper panel (labelled GUS) was probed with GUS sequences, stripped and reprobed with one-half of the 598bp inverted repeat sequence (middle panel, labelled 598bp). Lower portion of blot was cut out and probed with *DCL1* cDNA sequences as a loading control (lower panel, labelled DCL1). Lanes 1–5 contained DNA from F2 lines with empty donor sites; lane 6, ET500 parent. The up-shift in band size between ET500 parent and F2 progeny plants in the middle panel is because of destruction of a *Sac*1 site due to excision. Lanes 1, 2, 4, and 5; F2 progeny had lost *Ds* from the genome, but retained the 598bp fragment. Lane 3; F2 progeny had a reinsertion of *Ds* but retained the 598bp fragment at the original locus. (D) Southern blot analysis of *Sac*I digested DNA showing movement of the 598bp segment. Lanes 1 and 2 had F2 DNA from two independent F1 lines, and lane 3 had ET500 parental DNA. Probes were as labelled for panel (C). Lane 1 shows an F1 line in which the *Ds* and the 598bp inverted repeat had a change of position. Lane 2 shows a line that had lost *Ds* but not the 598bp inverted repeat sequence from the original location. Hybridization signals expected from the original location are identified with triangles; signals due to sequence alteration are identified with circles (loss of *Sac*I site adjacent to 598 bp repeat), asterisks denote movement of GUS and the 598bp repeat to another location.

The regions flanking the excision footprints (Figure 5 A, B) were examined by Southern analysis to determine the fate of the 598bp repeat after *Ds* excision (Figure 5C, D). For 43/47 independent events tested, the 598bp repeat remained at the empty donor site (Figure 5C, lanes 1–5). For 2/47 events it was lost from the donor site. Southern blot on F2 and sequencing through the ET500 insertion site on F3 (*LL* at nga8) confirmed the absence of *Ds* and the 598 bp repeat in these two cases. For 1/47 events, Southern analysis was consistent with a move of the repeat to another chromosomal position, not necessarily adjacent to a reinserted *Ds* (Figure 5D, lanes 1, 4). For the remaining one excision, the fate of the fragment was undetermined because results of Southern analysis and PCR were inconclusive.

## Discussion

We have shown that within the resolution limits of our measurement the excision in Arabidopsis of a *Ds* element from a homologous chromosome does not provoke interaction of the broken chromosome with its intact homologue, and that the repair mechanism resembles the immunoglobulin V(D)J joining reaction of vertebrates.

Previous experiments showed that at the *bronze* locus in maize *Ac/Ds* activity was not associated with homologous recombination [27]. The *bronze* locus, however, is a recombination hotspot [28], and thus could have been atypical in its behaviour with respect to *Ds* excision. Our observations in *Arabidopsis* together with previous observations in maize at *bronze* help generalize the conclusion that *Ds* excision does not frequently influence crossover recombination between homologous chromosome pairs. These results imply that although DNA transposable elements have evolved to provide evolutionary plasticity to the host genome through frequent rearrangements, their excision may seldom affect crossover of flanking arms of homologous chromosomes. We have shown that efficient excision of *Ds* element can occur despite the absence of canonical target site duplication at the 3′ end of the element. This was also reported for A*c* excision from the maize *P1*-vv varigated pericarp allele that lacks a target site duplication [24]. Finally, based on Southern analysis, the movement of non-*Ds* inverted repeat elements may be facilitated in *cis* by the activity of nearby transposable elements. Although we have not provided conclusive molecular evidence for movement of the repeat, our analysis is consistent with the recent finding that endogenous repetitive sequences are involved in a subset of chromosomal rearrangements mediated by *Ds* excision [29]. A possible mechanism of transposition of the 598 bp repeat could be cryptic transposase binding sites within this inverted repeat. When high-affinity transposase binds to the *Ds* TIRs, binding of further transposase molecules to nearby cryptic sites may be facilitated. Alternatively, DNA may occasionally slide through the bound transposase whereupon the enzyme makes a cleavage at a distance from the binding site (Fig 4). These interpretations suggest that the formation as well as further transposition of novel inverted repeat sequences are facilitated by existing transposable elements, and could contribute towards the evolution of new mobile elements. The alternative explanation, a move of the 598 bp repeat during a spontaneous rearrangement or recombination at the ET500 locus, not associated with Ds excision and repair, seems unlikely because the control cross ET500×wild type No-0 showed no such instability. More likely are the remaining two explanations: a transposition of the 598 bp repeat to a new chromosomal location, or a transposition during which the *Ds* and 598 bp repeat were rearranged within the ET500 locus. We suggest that the 598 bp repeat is mobile because we observed a loss of this repeat from the genome in association with two independent *Ds* transpositions.

In further support of the existence of cryptic binding sites within the 598 bp repeat, motifs were identified based on the results of Becker and Kunze [30] describing *Ac* transposase binding sites as ACG, TCG and A/TCGG in the 250 bp subterminal ends inside the 11-bp terminal IRs. We found these motifs 25 times in the 598 bp repeat. Consistent with previous observations [30] these motifs are present on both strands in direct and inverted repeat orientations. The number of motifs within the 598 bp repeat is comparable to that in *Ac* ends, namely, 25 in *Ac* 5′ and 20 in *Ac* 3′ TIRs, respectively. Although the motifs in the 598 bp inverted repeat are outside the terminal IR of the *Ds* element, flexibility of the transposase in DNA binding [30] may allow the transposase to ‘slide’ and recognize these cryptic sites while still requiring the TIRs of the *Ds* element in *cis* for transposition. The presence of transposase binding sites within DNA, however, is necessary but not sufficient for transposition [25].

The observed lack of detectable crossover enhancement by a pioneering transposable element in a foreign genome and the formation of novel repeated DNA sequences as a consequence of transposon movement have implications on the role of transposable elements in genomic sculpting. While we did not find any evidence of correlated crossover exchange at the site of excision, the influence of transposable elements as agents causing rare crossovers cannot be eliminated. Nevertheless, it appears that movements of transposons are unlikely to be the most important direct agent for causing somatic or germinal crossovers. On the other hand, the production of novel repeated elements might indirectly create the possibility of intra-chromosomal or inter-chromosomal crossover by homologous recombination in subsequent generations.

A consideration of the conserved and unique aspects of *Ds* footprints in maize [27], [31], [32], *Arabidopsis* [23; this work] and yeast [4], [21] suggest that binding of the transposase molecules at *Ds* TIRs may be sufficient for accurate excision of *Ds* but that the mechanism of repair of broken ends at the donor site is dependent on host factors. In maize and tobacco, repair occurs mostly by an error prone repair synthesis followed by end joining. In *Arabidopsis* and yeast, the major footprints are consistent with hairpin formation, hairpin opening and microhomology mediated end joining. One wonders whether by manipulating host factors in plants might the ends be forced to repair by homologous recombination as normally seen with P-element transposition in *Drosophila* [33], [34].

## Materials and Methods

**Strains and plant growth conditions**

*Arabidopsis thaliana* strain ET500 (La-er) was the source of *Ds* insertion element [19], and strain NaeAc (No-0) was the source of *Ac* transposase [35]. Conditions of growth and propagation were as described before [36].

### Verification of homozygous lines

ET500 and NaeAc putative homozygous insertion lines were selfed for two generations. In the second generation ET500 F2 segregated 100% (42/42) kanamycin resistant seedlings, and has GUS^+^ leaves with 100% penetrance and expressivity throughout the life cycle. In addition, a ring of cells on the ovary receptacle expressed GUS. The *GUS* transgene segregated as a dominant marker in the cross ET500×wild type Nossen: 31/42 of F2 progeny were GUS^+^, indicating a single dominant *GUS* transgene (*P*<0.005 for heterozygosity). Similarly, in the second generation of NaeAc selfing, 14/14 F2 plants were kanamycin resistant and tested positive for the transposase gene by PCR analysis. Additionally all 53 F1 plants of ET500×NaeAc scored positive for the *Ac* gene by PCR analysis. Sequencing of genomic DNA 301 bp to the left of the *Ds* insertion site and 341 bp to the right of the inverted repeat sequence showed 100% sequence homology between the Nae*Ac* and Landsberg *erecta* parental lines.

### Molecular methods

Southern blots were performed as described by Golden and colleagues [36]. Methods for DNA isolation, PCR, SSLP typing, cloning and analysis were described before [36], [37]. After amplification of SSLP markers, the PCR amplified DNA fragments were separated on a 4% Metaphor gel (FMC) at 65V for 4 hours. The polymorphic sizes are: nga8 (La-er 198 bp; No-0 168 bp); nga1111 (La-er 154 bp; No-0 146 bp or 150 bp); Ga1a/*Bsa*BI (La-er 0.707 kb and 0.527 kb; No-0 1.196 kb). PCR conditions for SSLP markers were: for nga1111 and Ga1a, 40 cycles of 15s (96°C)—15s (55°C)—30s (72°C); for nga8, 40 cycles of 15s (95°C)—15s (58°C)—30s (72°C). For amplifying *Ac* and *GUS* sequences, PCR condition was 36 cycles of 15s (95°C)—15s (58°C)—30s (72°C). The *Ac* primers were AC2210, 5′GTTGAGACATCATATGAGATC3′; and AC3080, 5′ATTGCAAGGCTAAGTATAGG3′. *GUS* primers were GUS43, 5′CTCGACGGCCTGTGGGCATTC3′; and GUS945, 5′GTGGTCGTGCACCATCAGCACG3′. PCR analysis of ET500 excision sites (footprints) used 36 cycles of 30s (96°C)—30s (63°C)—90s (72°C) (Primers were: 318E, 5′CAAGGTTGGATTCAGAGGAATCCTATTGTTCTTGAG3′; T6150-EC, GCTTACGTACATGGTCGATAAGAAAAGGCAATTTGTAGATG3′; and 58237E-2,

5′TAACAAGAATGAGATATAAAGTATCACCTTCTCATCATGAG3′). For PCR of excision sites in plants that lost both the Ds and ocs3′-RB (Primers: T6136, 5′CCATTCGCCCTATAGTGAGTCG3′; and T6409-2, 5′TGCCAGTCAGCATCATCACACC3′), the reaction condition was 36 cycles of 30s (96°C)—30s (63°C)—90s (72°C). PCR of the ocs3′-RB inverted repeat flanked by a portion of the 3′ *Ds* and plant DNA was with 0.25 µM each of primers Ds3-2E (5′CGTTACCGGTATATCCCGTTTTCG3′) and 58179E (5′CAATGCTCTCTGCCTTCGCCGTTAAAG3′) for 36 cycles of 30s (96°C)—30s (61.7°C)—120s (72°C).

PCR of the 598 bp inverted repeat flanked by 3′*Ds* and plant genomic DNA was performed in a reaction volume of 50 µl with 1M betaine (Sigma) and 6.25 units *Taq* DNA polymerase (Qiagen), with 0.25 µM each of primers Ds3-2E and 52879E. The reaction was a ‘hot start’ with 1 cycle of denaturation at 95°C, then 36 cycles of 30 s (96°C)—30s (61.7°C)—2min (72°C).

### Single-stranded PCR and cloning of DNA flanking the 3′ and 5′ *Ds*

Thermal asymmetric interlaced (TAIL) PCR [38] for DNA genomic flanking the 5′ *Ds* in ET500 was accomplished with primers: Ds5-1, Ds5-2, Ds5-3 and AD3, with secondary and tertiary cycles as described [39]. DNA on the 3′ flank of *Ds* was cloned by single-stranded PCR base on the “5′ Race System for Rapid Amplification of cDNA Ends, Version 2.0” (GibcoBRL), with modifications as follows: single-stranded PCR of 1 µl of ET500 genomic DNA with Ds3-1 and *Taq* Polymerase High Fidelity as follows: 1 cycle 94°C for 2 min; 40 cycles of 15 sec at 94°C, 15 sec annealing at 55°C and 1 min extension at 68°C; PCR of C-tailed DNA, Ds3-2 with 20 sec denaturation at 94°C, 15 sec annealing at 50°C and 3 min polymerization at 68°C; 40 cycles; nested PCR with CUDs3-3A, 5′CAUCAUCAUCAUGGTAGAGGTATTTTACCGACC3′, 94°C hot start 20 sec at 94°C, 15 sec at 55°C and 3 min at72°C, 40 cycles. Polymerization for the 40th cycle was extended from 3 to 7 min.DNA fragments cloned from the 3′ flank representing one-half of the inverted repeat were used probing Southern blots. PCR primers for probes were T6136 and T6409-2. Primer T6136 corresponds to 22 bases immediately (3′) of the *Sac*I site, within the inverted repeat, as shown in Fig. 1 (The 5′ end of primer T6409-2 is complementary to base 6409 on the ET500 parental T-DNA vector; Genbank accession number M35007.1), and its 3′ end is complementary to base 6378 on the same T-DNA sequence. The 5′ end crosses the point of inversion by three bases (Fig. 1). Sequencing of the Nae*Ac* and wt Landsberg *erecta* parental genomic DNA flanking the ET500 insertion site for comparison of homology was accomplished with primers 318E and 58179E.

### DNA Sequencing

Sequencing reactions were as stated in the ABI Prism BigDye Terminator cycle Sequencing Ready Reaction Kit (Applied Biosystems), with 16 pmoles of primer and 6ul Big dye Terminator Ready Reaction Mix (Applied Biosystems) for a 20 ul reaction. Sequencing primers and PCR primer sequences were the same. To sequence the 598 bp inverted repeat with flanking *Ds* and plant DNA, the reaction was 40 µl with 32 pmoles Ds3Sac, 5′CCGTTTTCATCCCTAGAGCTCCAATTCGC3′, 1M betaine (Sigma) 16 µl Big Dye Terminator Ready Reaction Mix, 1M betaine as follows: (98°C)-3min, then 25 cycles of 30s (96°C)—4min (64.0°C).
